# Time-series NARX feedback neural network for forecasting impedance cardiography ICG missing points: a predictive model

**DOI:** 10.3389/fphys.2023.1181745

**Published:** 2023-06-06

**Authors:** Sara Benouar, Malika Kedir-Talha, Fernando Seoane

**Affiliations:** ^1^ Department of Clinical Science, Intervention and Technology, Karolinska Institutet, Stockholm, Sweden; ^2^ Laboratory of Instrumentation, Department of Instrumentation and Automatics, Institute of Electrical Engineering, University of Sciences and Technology Houari Boumediene, Bab Ezzouar, Algeria; ^3^ Department of Medical Technology, Karolinska University Hospital, Stockholm, Sweden; ^4^ Department of Clinical Physiology, Karolinska University Hospital, Stockholm, Sweden; ^5^ Department of Textile Technology, University of Borås, Borås, Sweden

**Keywords:** artificial neural networks, NARX, impedance cardiography, machine learning, time-series predictive model, characteristic point detection

## Abstract

One of the crucial steps in assessing hemodynamic parameters using impedance cardiography (ICG) is the detection of the characteristic points in the dZ/dt ICG complex, especially the X point. The most often estimated parameters from the ICG complex are stroke volume and cardiac output, for which is required the left ventricular pre-ejection time. Unfortunately, for beat-to-beat calculations, the accuracy of detection is affected by the variability of the ICG complex subtypes. Thus, in this work, we aim to create a predictive model that can predict the missing points and decrease the previous work percentages of missing points to support the detection of ICG characteristic points and the extraction of hemodynamic parameters according to several existing subtypes. Thus, a time-series non-linear autoregressive model with exogenous inputs (NARX) feedback neural network approach was implemented to forecast the missing ICG points according to the different existing subtypes. The NARX was trained on two different datasets with an open-loop mode to ensure that the network is fed with correct feedback inputs. Once the training is satisfactory, the loop can be closed for multi-step prediction tests and simulation. The results show that we can predict the missing characteristic points in all the complexes with a success rate ranging between 75% and 88% in the evaluated datasets. Previously, without the NARX predictive model, the successful detection rate was 21%–30% for the same datasets. Thus, this work indicates a promising method and an accuracy increase in the detection of X, Y, O, and Z points for both datasets.

## 1 Introduction

The impedance cardiography (ICG) signal (dZ/dt) is a result of the first-time derivative of the impedance changes signal ∆Z in the thorax area. Inspired by PQRST of the electrocardiogram (ECG) signal, the dZ/dt signal is annotated by seven typical characteristic points, namely ABEXYOZ (Lababidi et al., 1970).

Some of the most difficult points to detect in the dZ/dt signal, especially in automatic detection, are the X and B points.

Those characteristic points are crucial for calculation of the most common hemodynamic metrics for assessing cardiac function, such as the left ventricular pre-ejection time (LVET), which is a precursor for the calculation of stroke volume (SV). LVET is directly associated with the systole period of the cardiac cycle (Wang and Gottlieb, 2006; Cybulski, 2011). It is also one of the first analyzed indices in the literature (Kizakevich et al., 1993; Sherwood et al., 1998). It is defined as the interval period between the B and X points in the dZ/dt waveform (Bernstein and Lemmens, 2005).

The SV is defined as the amount of blood ejected from the left ventricle in one cardiac cycle. One of its coexisting parameters is cardiac output (CO), which is the total amount of blood pumped in 1 min. Thus, CO results in the product of SV and heart rate (HR).

In addition, many other indices are related to the detection of the dZ/dt characteristic points, such as the isovolumic relaxation time (IVRT), which is defined as the period interval between X and O points. It measures the activity of ventricular relaxation related to diastolic function (Summers et al., 2003).

Thus, the dZ/dt characteristic points are crucial elements in the calculation of LVET, SV, and other important hemodynamic parameters. However, reliable detection of those points remains difficult, especially in automatic processing (Sherwood et al., 1990; DeMarzo and Lang, 1996; Meijer et al., 2008). Moreover, straightforward detection of the other characteristic points of the dZ/dt waveform encounters similar problems (Sherwood et al., 1990).

### 1.1 Detection of ICG characteristic points

**FIGURE 1:**
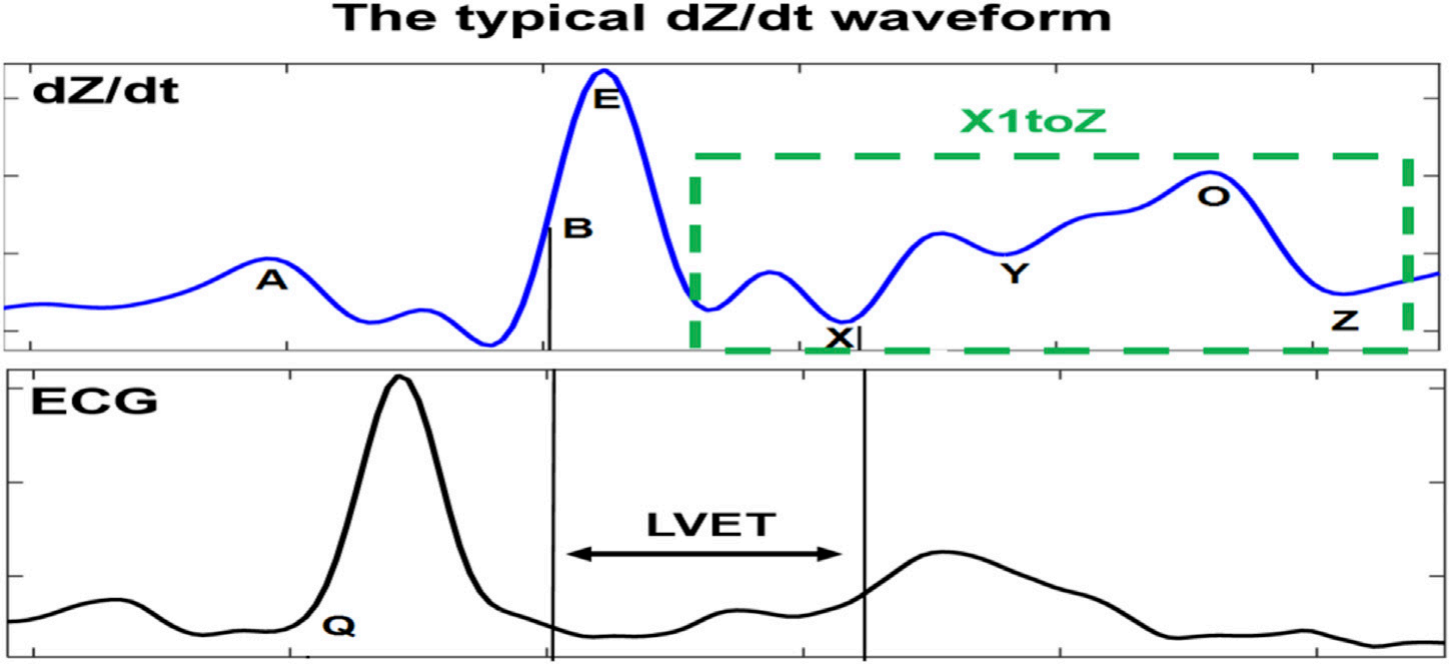
Typical dZ/dt waveform recorded simultaneously to the ECG signal. The dZ/dt signal is marked by its characteristic points. LVET is the interval between B and X points. The main differences of atypical dZ/dt complexes (subtypes) are principally in the X1–Z part, where X1 is the minimum point on the descending slope of the E-wave.

**FIGURE 2:**
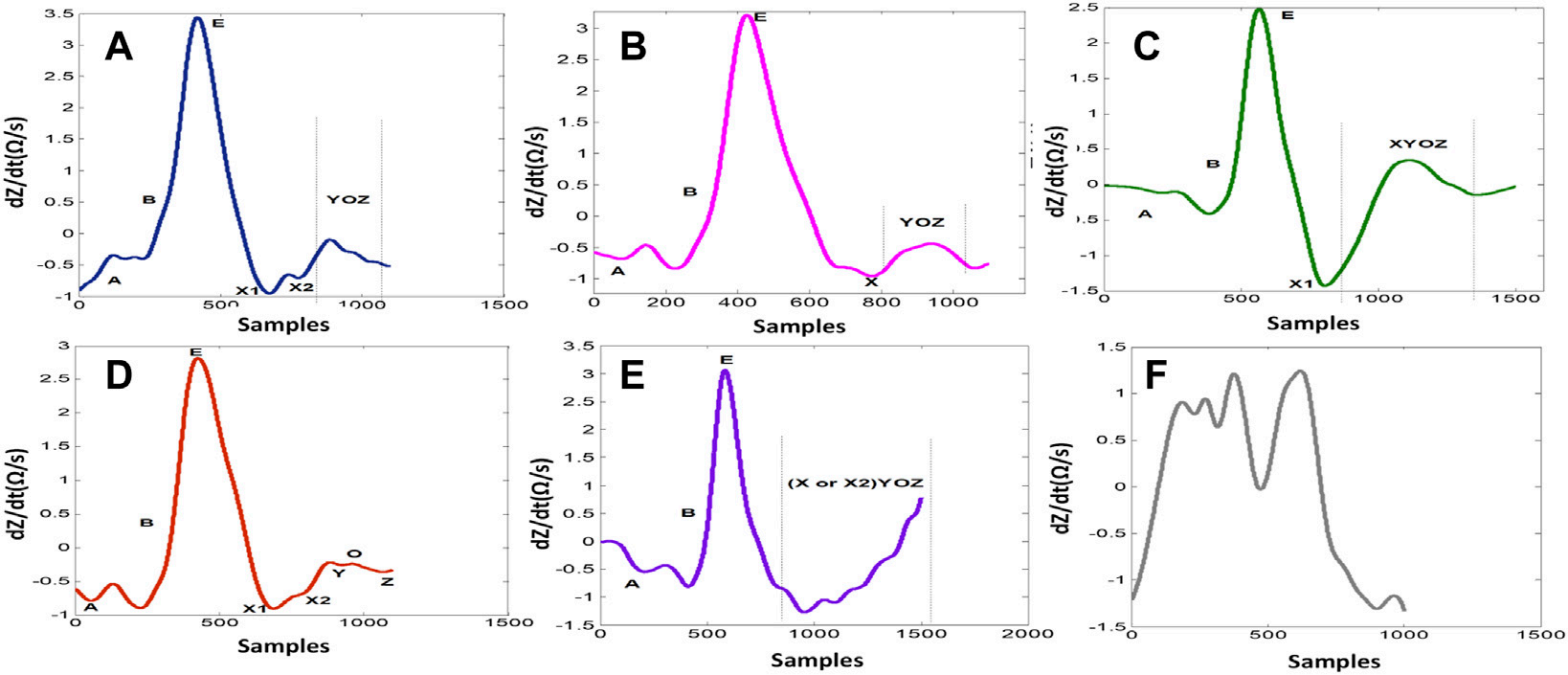
dZ/dt waveform subtypes. **(A)** ABEXYOZ3. **(B)** ABEXYOZ1. **(C)** ABEXYOZ4. **(D)** ABEXYOZ2. **(E)** ABEXYOZ5. **(F)** ABEXYOZu.

One of the main challenges is the observed changes in the ICG waveforms. These changes have been interpreted in different ways and are usually left with unclear conclusion on their sources (Kööbi et al., 2003; DeMarzo, 2009; Cybulski, 2011; Tronstad et al., 2019). The observed variability between waveforms impacts the accurate detection of specific ICG points, such as point X, and this has led to the proposal of different detection mechanisms (BIOPAC Systems Inc, 2018). A derivative of the ICG signal, dZ/dt, as shown in Figure 1, was used to accurately detect the ejection time (Patterson and Shewchun, 1964; Kubicek et al., 1966). The authors demonstrated that the maximum point of the first derivative, dZ/dtmax (traditionally noted as point C or E), is related to the rate of ventricular blood ejection (Kubicek et al., 1970). Additionally, to avoid interference in the baseline, the crossing point with the baseline of the dZ/dt signal (noted as the B point) (Sherwood et al., 1998) (opening of the aortic valve) was changed to a 15% response of the dZ/dt waveform from the baseline (Kizakevich et al., 1993). Another method exists for calculation of the B point around the R peak of the ECG signal using an equation. However, there is no golden standard algorithm, and, usually, the assembling average method (Riese et al., 2003) is the standard process used to calculate the hemodynamic parameters from the dZ/dt signal. To attenuate the changing morphology, however, averaging all beat-to-beat cycles together may result in discarded information that is potentially relevant for ICG analysis. It also includes a higher level of missing points (Benouar et al., 2018). In a previous study (Benouar et al., 2018), systematic variability was reported in the dZ/dt signals, which resulted in five distinct ABEXYOZ complex subtypes. Not all subtypes include typical ICG characteristic points. Figure 1 presents a typical ABEXYOZ ICG waveform. Figure 2 presents a reminder of the different existent ICG subtypes, and more details are explained elsewhere (Benouar et al., 2018). In this work, an overview of the first steps toward the detection of the ICG characteristic points according to the classified ABEXYOZ subtypes is presented, where a predictive model has been trained and tested on two different datasets V and S. The R peak of the ECG has been used to create a time series between the R-ECG and the ICG characteristic points (ABEXYOZ), in addition to those specified by the ABEXYOZ subtypes (X1 and X2). Thus, to widen the possibility of evaluating LVET and other ICG parameters according to the ABEXYOZ subtypes, automatic detection and prediction of the ICG characteristic points are required for further investigation of the characteristic points and ICG index parameters.

To this end, a non-linear autoregressive model with exogenous inputs (NARX) is used for predicting the future values of the ICG characteristic points, including detection of the suitable X point. The learning of such long-term temporal dependencies using several existing algorithms is critical, and the algorithm with the best performance was chosen in this study.

## 2 Materials and methods

### 2.1 ICG datasets

In this work, two completely different datasets were used (named dataset V and dataset S). The measurements contained in the datasets were recorded at the Laboratory of Medical Textile-Electronics at the University of Borås, Sweden, and have been reported elsewhere (Rempfler, 2011; Marquez et al., 2013; Hafid et al., 2017; Hafid et al., 2018).

The ICG recordings were completed after participants signed an informed consent form according to ethical approval 274-11 granted by the Regional Committee for Ethical Vetting of Gothenburg. As indicated in Table 1, more than 700 s containing more than X ICG complexes from a total of eight volunteers are contained in two different datasets.

**TABLE 1:** Description of datasets V and S.

	Nr electrodes	Nr subjects	Device	Total recording length s)	Sampling frequency (Hz)
Dataset V	8	4	Z-RPI	480	250
Dataset S	8	4	Respimon	240	1,000

The recordings were obtained with two different ICG recorders using different but compatible electrode positions on several different volunteers. The sampling frequency was 250 Hz for dataset V and 1,000 Hz for dataset S (see Table 1).

The recordings were carried out with the following:

• A Respimon impedance recorder (Medical Electronics Lab, Chalmers University of Technology, Sweden) as described by Rempfler (2011) and Marquez et al. (2013). Electrode placement of both datasets V and S of eight electrodes is described by Benouar et al. (2018).
• All volunteers were healthy and young.
• And data were fully anonymized.

**FIGURE 3:**
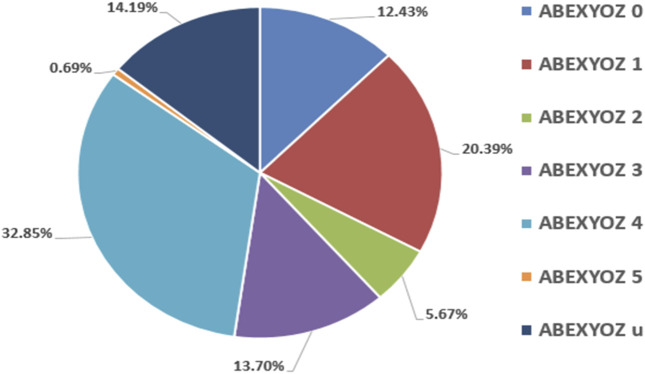
Percentages of ICG subtypes in both datasets V and S.

Figure 3 presents the percentages of ICG complex subtypes, also known as ABEXYOZ complex subtypes in both datasets V and S, where an imbalance between subtypes and dominance of some subtypes against others is observed.

**FIGURE 4:**
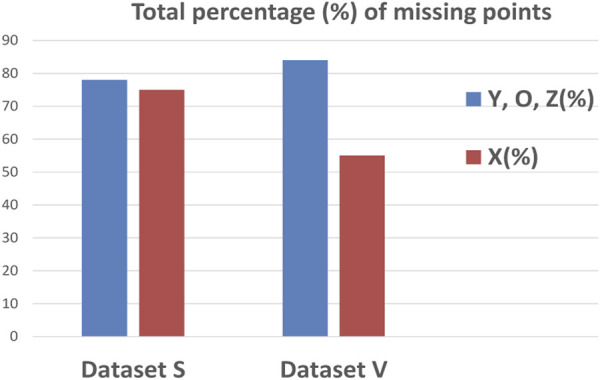
Percentages of missing ICG characteristic points for both datasets V and S.

Figure 4 represents the percentages of missing points in datasets S and V, where most missing points are crucial ones for calculation of the most common hemodynamic parameters. Some ICG characteristic points have the same behavior; they appear and disappear together, such as the ABE points and YOZ points, suggesting that the variability of the ICG waveform can affect more than one characteristic point.

### 2.2 Data processing for NARX ingestion

The main purpose for building a NARX predictor was principally to create a model that can predict the position of ICG characteristic points in ABEXYOZ complex subtypes with missing points, and to decrease the actual percentages of missing points (Benouar et al., 2018).

**FIGURE 5:**
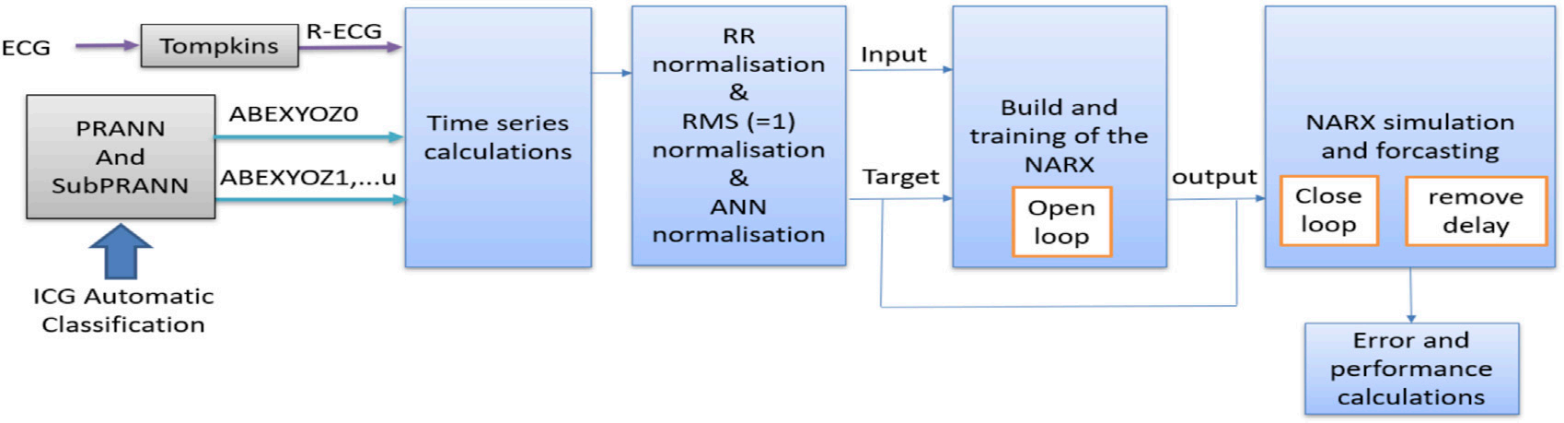
Flowchart of general data processing implemented in this study.

Figure 5 represents the general process for data processing in NARX ingestion. All the steps are detailed in the following subsections.

#### 2.2.1 Collecting the data

The data used in this part of the work were from datasets V and S (Benouar et al., 2018).

**FIGURE 6:**
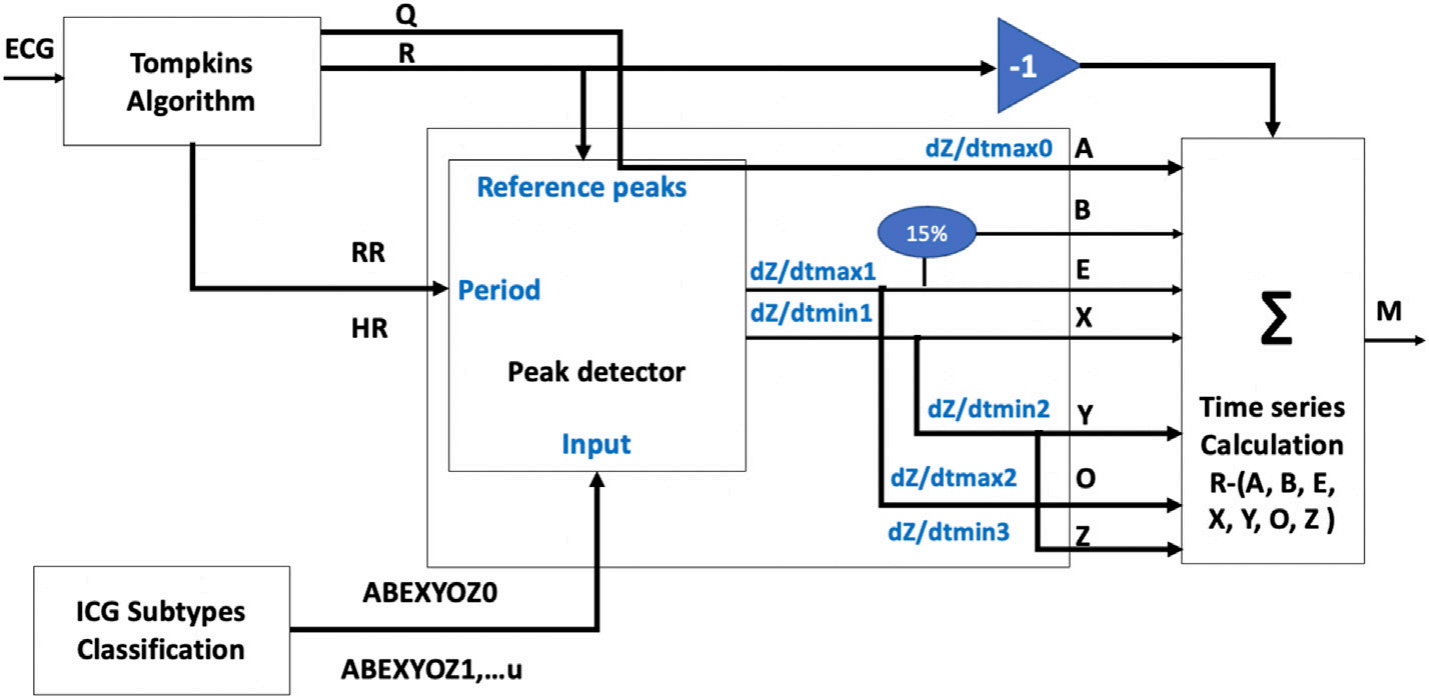
Flowchart of time-series calculation.

Thus, a time-series table was calculated for each subtype of each volunteer, which were the time intervals between the R peaks of the reference signal ECG and the ICG characteristic points for each ABEXYOZ complex, as shown in Figure 6. This resulted in a matrix M with the time series as columns: RR, RA, RB, RE, RX1, RX2, RY, RO, and RZ, knowing that

• X1 is the first minimum of the ascending slope after the E point
• X2 is a true X in ABEXYOZ0 and ABEXYOZ1, with X missing elsewhere

And the rows include all the volunteers’ subtype cycles for both datasets.

• The absence of some subtypes in some volunteers is noted as NaN values, where they were replaced by the mean of the other cycles that were available

#### 2.2.2 Preparing the data

The data were processed as follows (see the normalization block in Figure 5):

1. Normalizing the columns by the RR: To account for differences in heart rate between subjects, we normalized the data by dividing each value in the dataset by the corresponding RR interval.
2. It was noticed that each acquisition system [Z-Rpi and Respimon (Rempfler, 2011; Marquez et al., 2013; Hafid et al., 2017; Hafid et al., 2018)] has its own range of data; therefore, each acquisition system should have its own predictive model, which is not practical. Thus, the data were normalized between the two datasets in order to have one model that fits all the data. To preserve the relationships between the components of the value concerning the length, the sum of squares normalization method was used at this stage to scale the data so that each column has a sum of squares equal to 1, ensuring equal weight was given to each variable during analysis. This was to prevent certain features from dominating the analysis and enables comparison of data across different datasets.
3. Preparing the format of the data according to the chosen model: In our case, as a standard neural network matrix of cell arrays, we converted the normalized matrix into a cell array where each row of the cell array corresponds to a single observation, and the columns contain the features.

### 2.3 Time-series NARX feedback neural networks

The time-series feedback neural network NARX is a recurrent neural network architecture, and it can be used in several applications as apredictor,non-linear filter,modeling non-linear dynamic systems.

**FIGURE 7:**
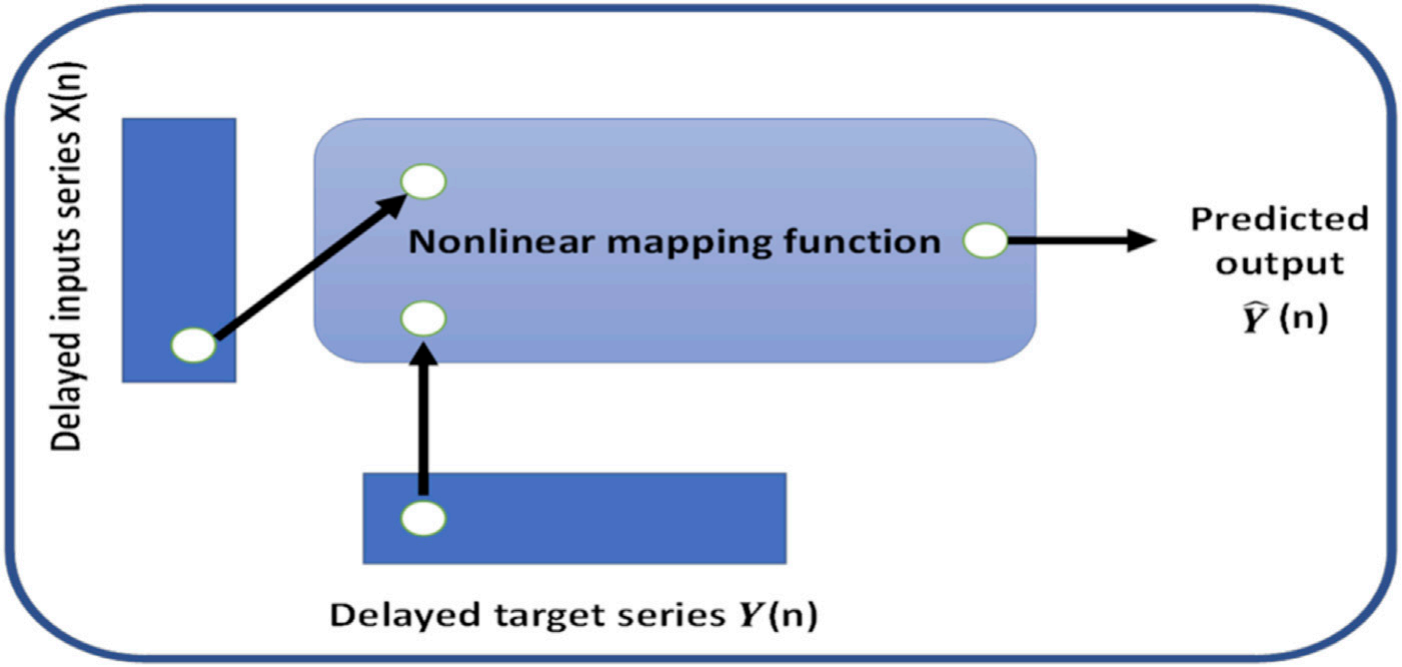
General architecture of the NARX model.

In this work, the NARX was used as a predictor to estimate the next value of the input signal; *i.e.*, the NARX was used as a time series non-linear prediction model. Figure 7 presents the general architecture of the NARX model.

The chosen method was suitable for time-series prediction and modeling using a dynamic recurrent artificial neural network. This form of prediction is known as non-linear autoregressive (NAR) and fits our application. The autoregressive function can be written as follows:

$$y\left(t\right)=f\left(y\left(t-1\right),\ldots ,y\left(t-d\right)\right).$$ (1)

Our configuration was set with eight inputs and eight outputs. The inputs correspond to the intervals between ICG characteristic points ABEXYOZ and R-ECG. Two possible values of X (X1 or X2) presented previously by the ICG are noted as follows: RA/RR, RB/RR, RE/RR, RX1/RR, RX2/RR, RY/RR, RO/RR, and RZ/RR.

In this work, the basic default setting model, which consists of one layer input of ten neurons with delay *d* = 2, was used.

**FIGURE 8:**
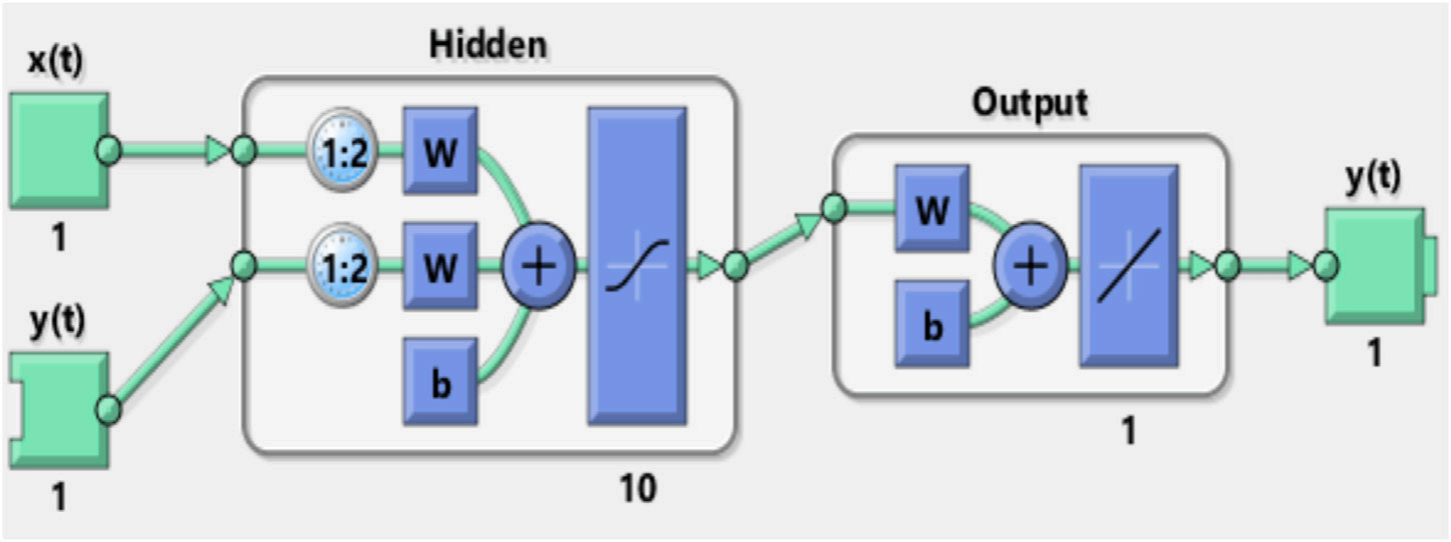
Synoptic schematic of the NARX prediction model during the training process.

**FIGURE 9:**
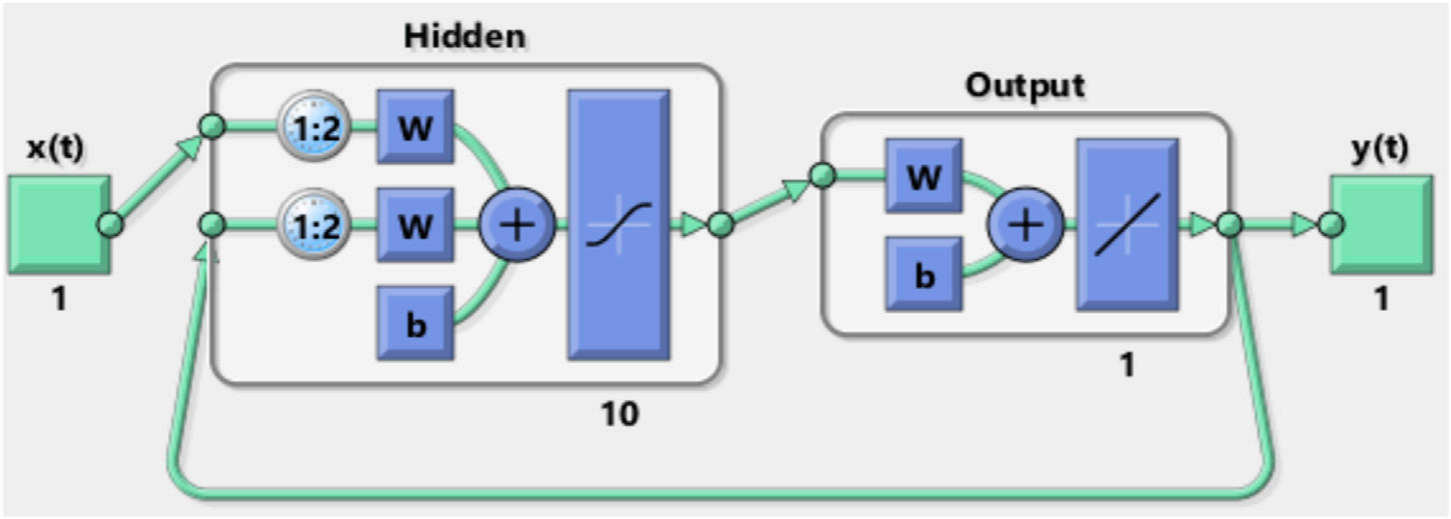
Synoptic schematic of the NARX prediction model during the testing process.

Figures 8, 9 show the NARX schematic during the training and testing process, respectively.

#### 2.3.1 Training the model

The training was carried out as follows:

1) The model was trained using the typical ICG cycles (labeled as ABEXYOZ0) because we aimed to use the model to predict the right values of missing points in the rest of the ABEXYOZ subtypes that have at least one or several missed points. Validation and test training were carried out as follows.

The input vectors and target vectors were randomly divided into three sets.

- 70% was used for training
- 15% was used to validate that the network was generalizing and to stop training before overfitting
- The last 15% was used as a completely independent test of network generalization
2) The training was carried out with the open-loop prediction (see Figure 8) because it is more efficient than with closed-loop prediction. Thus, this allows us to supply the network with correct feedback inputs by training it to produce the correct feedback outputs.
3) The training was carried out several times using several algorithms until the best performance was reached (Levenberg–Marquardt, scaled conjugate gradient, and Bayesian regularization).

Once training was completed, the loop was closed for multi-step prediction tests and simulation.

## 3 Results

### 3.1 Selection of the training model evaluating training performance and predictive accuracy

Table 2 presents the Pearson correlation coefficient (R), the root means squared error (RMSE), the mean absolute error (MAE), the mean square error (MSE), and the cross-validation (VEcv) obtained for the three different algorithms: Levenberg–Marquardt, Scaled Conjugate Gradient, and Bayesian regularization. Such parameters were used to evaluate the accuracy and performance of the predictive models.

**TABLE 2:** Accuracy parameters of the three different algorithms: Levenberg–Marquardt, scaled conjugate gradient , and Bayesian regularization.

	VEcv (%)	MSE	RMSE	MAE	R
LV	76	0.001	0.029	0.023	0.98
SCG	72	0.004	0.059	0.037	0.96
BR	74	0.002	0.039	0.030	0.98

Our results are expressed through time series prediction regression plots, where the letter “R” typically refers to Pearson’s correlation coefficient, which is a measure of the strength of the linear relationship between the targeted and predicted output of the model. This correlation coefficient is used to assess the goodness of fit of a linear regression model for a time series prediction model.

**FIGURE 10:**
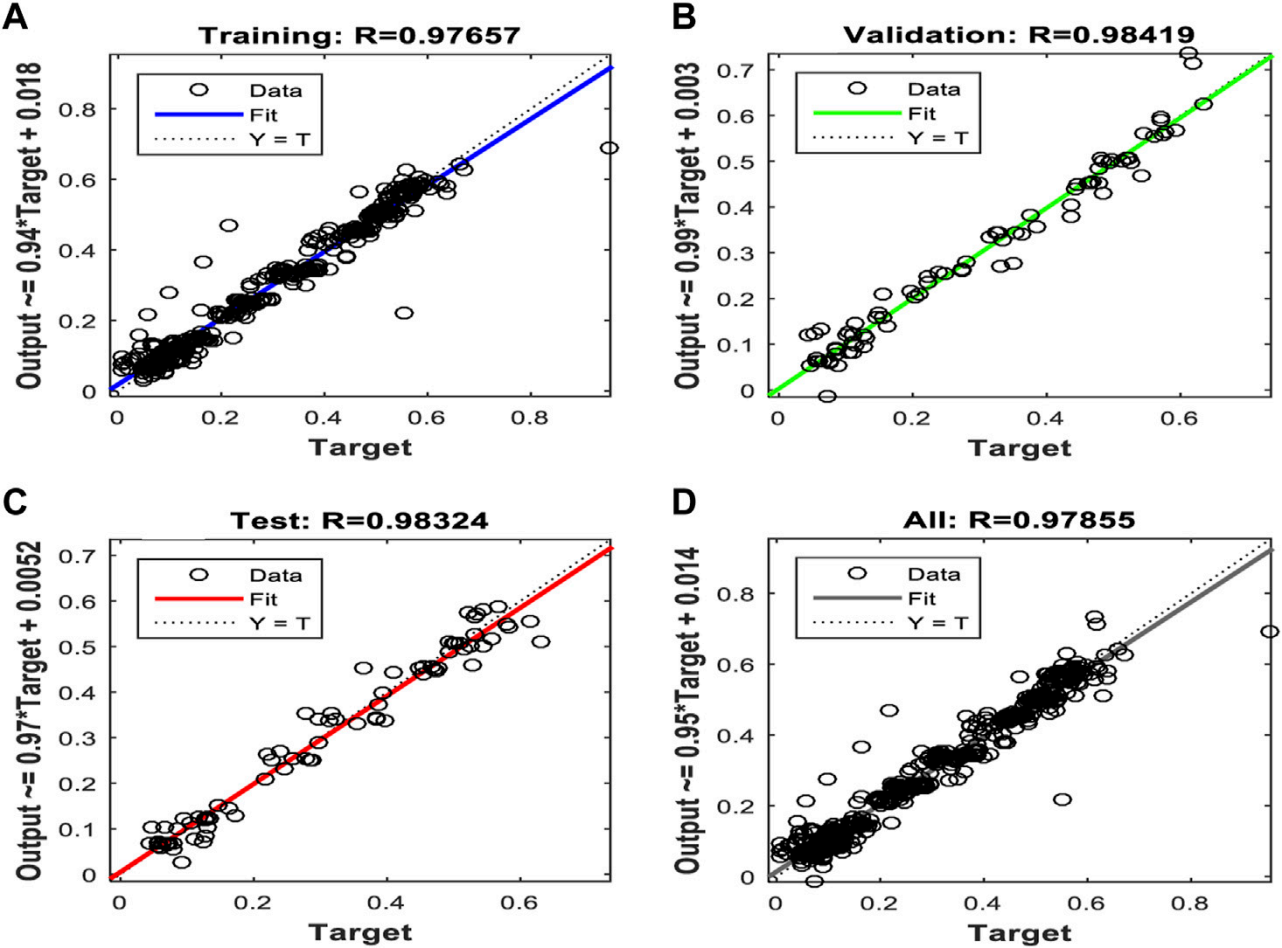
Regression plots of the model trained with the Levenberg–Marquardt algorithm. **(A)** Training **(B)** Validation **(C)** Test **(D)** All.

**FIGURE 11:**
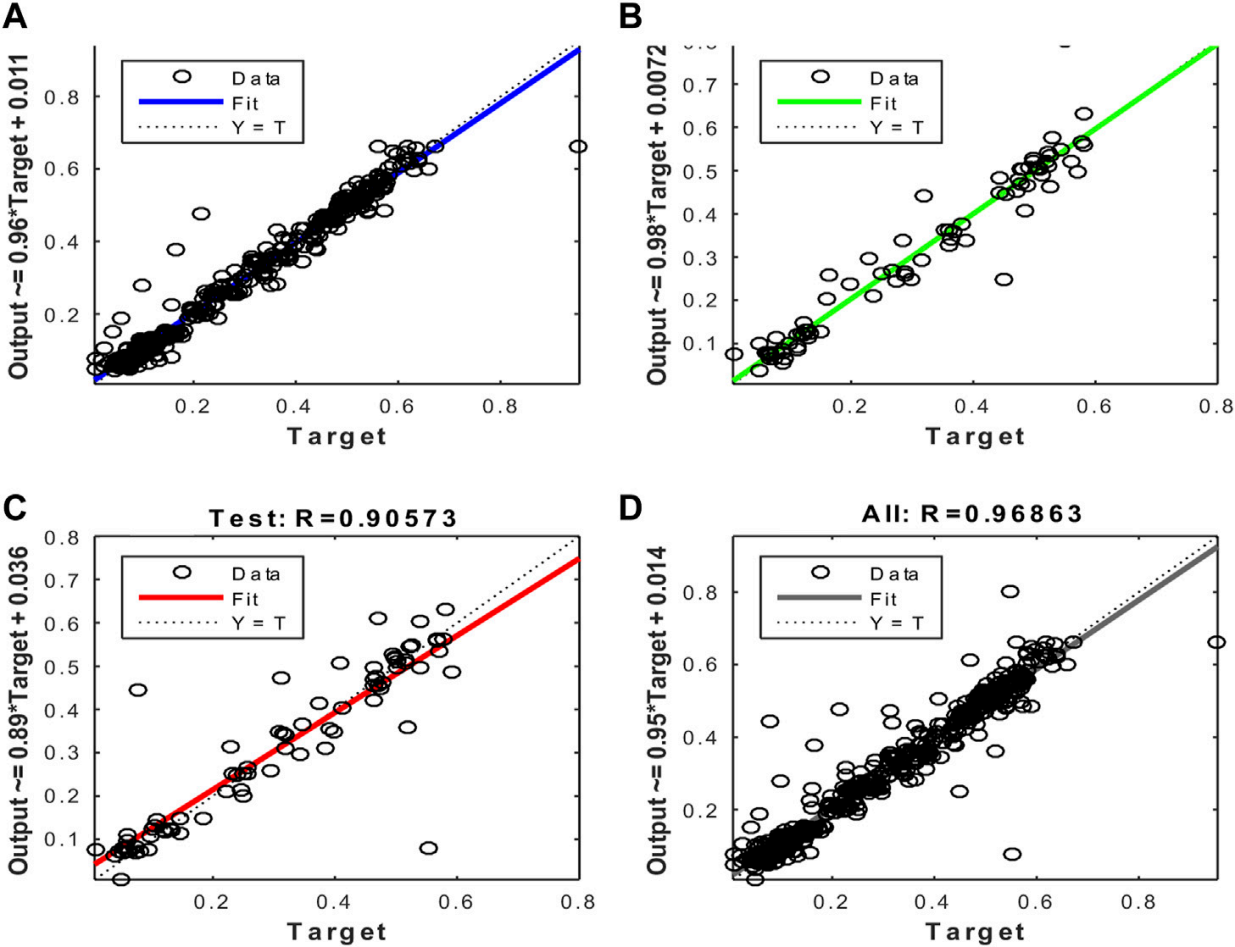
Regression plots of the model trained with the Scaled Conjugate Gradient algorithm. **(A)** Training **(B)** Validation **(C)** Test **(D)** All.

**FIGURE 12:**
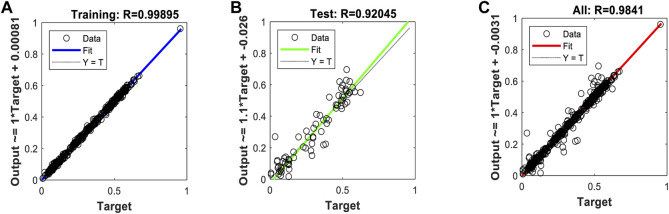
Regression plots of the model trained with the Bayesian regularization algorithm. **(A)** Training **(B)** Validation **(C)** All.

Figures 10, 11, and 12 show the performance results of the training of the model using the three evaluated algorithms, Levenberg–Marquardt, Scaled Conjugate Gradient, and Bayesian regularization, respectively.

After evaluating the obtained training performance and predictive accuracy, the training algorithms were ranked as follows: first Levenberg–Marquard, second Bayesian regularization, and third scaled conjugate gradient.

Thus, the chosen model was the model trained with the Levenberg–Marquardt algorithm. The performance achieved when forecasting the missing characteristic points through all the ABEXYOZ subtypes was similar across the S and V datasets, both producing an R value = 0.93. While dataset V had a more consistent performance with R = 0.9 or above, dataset S exhibited the best performance of all cases for subtypes ABEXYOZ2 and ABEXYOZ5, with R = 0.99. The worst performance was obtained from dataset V, producing R = 0.82 for ABEXYOZ3.

### 3.2 Forecasting the performance of the implemented NARX

Tables 3, 4 present the accuracy parameters of the target and the output (predicted values). The performance of the model was evaluated for each dataset separately and all together. In addition to the regression coefficient (R), RMSE and MAE were used to evaluate the model accuracy of our neural network. Moreover, MSE and the VEcv were calculated to evaluate the accuracy of the NARX model during and after the training (Table 3). The results are presented per subtype in Table 4. The equations of the accuracy parameters are presented in Table A1.

**TABLE 3:** Accuracy parameters of the detection of the ICG characteristic points during the training and forecasting stages.

Training; open loop		VEcv (%)	MSE	RMSE	MAE	R
All		76	0.001	0.036	0.023	0.98
Dataset S		73	0.004	0.053	0.034	0.96
Dataset V		73	0.004	0.062	0.038	0.95
**Forecasting**		**VEcv (%)**	**MSE**	**RMSE**	**MAE**	**R**
ALL	**Closed loop**	72	0.004	0.064	0.044	0.95
	**Removed delay**	**74**	**0.003**	**0.052**	**0.034**	**0.96**
Dataset S	**Closed loop**	73	0.003	0.055	0.042	0.96
	**Removed delay**	**75**	**0.001**	**0.034**	**0.025**	**0.98**
Dataset V	**Closed loop**	79	0.005	0.071	0.050	0.93
	**Removed delay**	**72**	**0.004**	**0.062**	**0.044**	**0.95**

Bold values indicate the improved forecasting results.

**TABLE 4:** Accuracy parameters of the detection of forecasting the ICG characteristic points after removing the delay results per subtypes.

		VEcv (%)	MSE	RMSE	MAE	R
ABEXYOZ1	Dataset S	73	0.003	0.056	0.043	0.97
	Dataset V	70	0.004	0.065	0.040	0.92
ABEXYOZ2	Dataset S	76	0.001	0.030	0.021	0.99
	Dataset V	75	0.001	0.031	0.022	0.98
ABEXYOZ3	Dataset S	55	0.019	0.100	0.096	0.82
	Dataset V	65	0.008	0.093	0.056	0.91
ABEXYOZ4	Dataset S	59	0.011	0.098	0.088	0.89
	Dataset V	63	0.008	0.094	0.058	0.90
ABEXYOZ5	Dataset S	76	0.001	0.032	0.020	0.99
	Dataset V	75	0.003	0.055	0.026	0.97

### 3.3 Enhancement in the detection of XYOZ

Compared to Figure 4, Table 5 shows an enhancement in the accuracy percentages of the detection of ICG characteristic points XYOZ across the different ABEXYOZ complex subtypes.

**TABLE 5:** Accuracy percentages of the detection of ICG characteristic points in the different ABEXYOZ complexes.

Dataset S	ABEXYOZ1	ABEXYOZ2	ABEXYOZ3	ABEXYOZ4	ABEXYOZ5
X (%)	78	79	74	76	79
YOZ (%)	78	78	74	76	78
**Dataset V**	**ABEXYOZ1**	**ABEXYOZ2**	**ABEXYOZ3**	**ABEXYOZ4**	**ABEXYOZ5**
X (%)	52	55	51	51	54
YOZ (%)	82	84	82	82	83

Bold values indicate the improved forecasting results.

The values for Lin’s concordance correlation coefficient shown in Table 6 indicate that the accuracy of the prediction was better for subtypes ABEXYOZ1 and ABEXYOZ2 than for subtypes ABEXYOZ3 and ABEXYOZ4.

**TABLE 6:** Lin’s concordance correlation coefficient of the predictive model for forecasting the missing characteristic points in all the ABEXYOZ subtypes for each dataset.

Lin’s (CCC)	ABEXYOZ1	ABEXYOZ2	ABEXYOZ3	ABEXYOZ4	ABEXYOZ5
Dataset S	0.90	0.96	0.67	0.69	0.96
Dataset V	0.89	0.91	0.78	0.73	0.93

**FIGURE B1:**
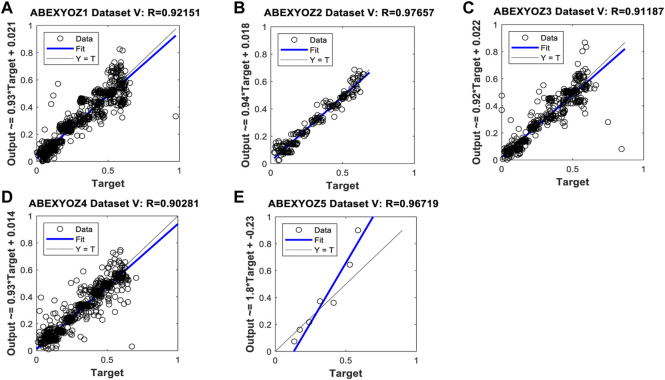
Regression plot result examples for forecasting missing points in ABEXYOZ subtypes in dataset V.

**FIGURE B2:**
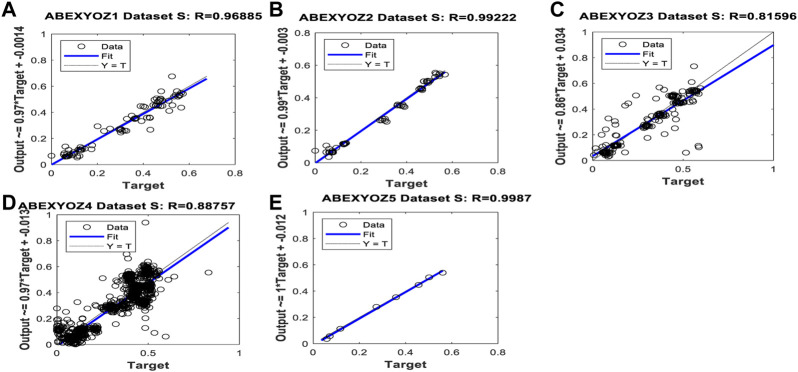
Regression plot result examples for forecasting missing points in ABEXYOZ subtypes in dataset S.

When focusing on the linearity of the NARX and evaluating the regression plots obtained in Figures B1, B2, we can see that the linearity of the predicted values is remarkable, providing an R coefficient ranging from 0.90 for ABEXYOZ 4 to 0.97 ABEXYOZ2 in dataset V. ABEXYOZ2 exhibited the best forecasting performance (see Figure B2B), with almost the same accuracy in the training stage (Figure 12A), which is a promising result in forecasting the right X point, while ABEXYOZ3 presented the lowest accuracy with R = 0.81 (Figure B2C).

### 3.4 Identification of the most suitable X point in complexes with multiple candidates

**FIGURE 13:**
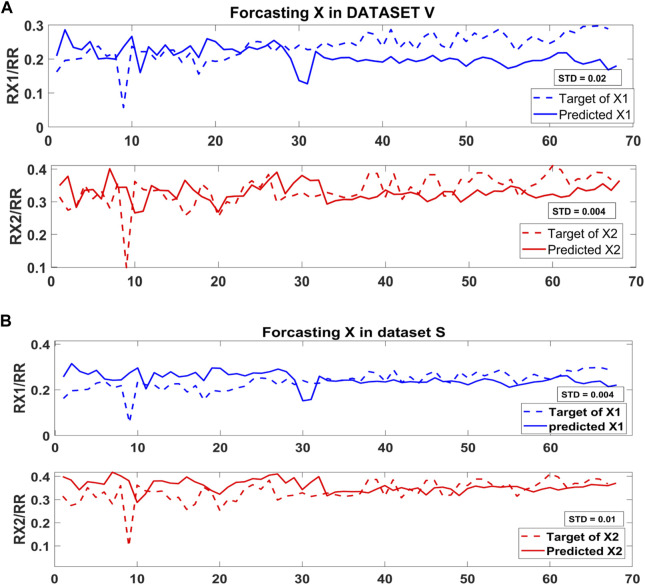
Standard deviation forecasting the X point in **(A)** dataset V and **(B)** dataset S.

As mentioned previously, the ICG subtype ABEXYOZ2 presents two possible true X (X1 or X2). Figure 13 presents the forecasting of X1 and X2 in subtype ABEXYOZ2 for both datasets, and it shows that X1 provided a smaller standard deviation regarding the reference ratio used as reference X1/RR for dataset S, while X2 provided a smaller standard deviation for dataset V.

### 3.5 Missing points

**FIGURE 14:**
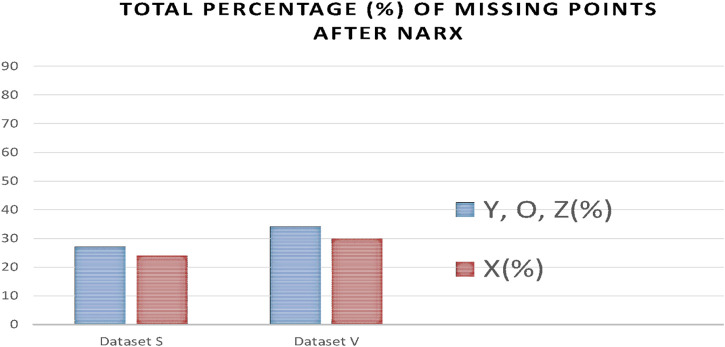
Percentages of missing ICG characteristic points after applying the NARX to each dataset V and S.

As a result of processing the time series with the NARX predictor, the number of missing points was significantly reduced. Figure 14 indicates a reduction of missing points from 76% and 56% to 23% and 29% for X points in dataset S and V, respectively, and from 79% and 83% to 27% and 33% for Y, O, and Z points in datasets S and V, respectively.

## 4 Discussion

### 4.1 Selecting the training model

First, an evaluation of the algorithms was conducted to choose the algorithm that performs with higher accuracy on the datasets used for this specific application. All algorithms showed a good performance range with a maximum VEcv of 76% at LV and a minimum of 72% at SCG. Regardless of the R values of 98 for both LV and BR, the LV was chosen for having a higher VEcv and lower MSE, RMSE, and MAE than the BR (see Table 2). Thus, the chosen algorithm was Levenberg–Marquardt, and the two other algorithms are Bayesian regularization and scaled conjugate gradient; this latter algorithm uses less memory, so it might be a suitable choice if the hardware selected for implementation presents computation power limitations. In such a case, there will be a trade-off between accuracy and memory.

The training starts with an open loop to feed the network with the targeted information; for this, typical ICG subtypes have also been used to train our model to detect typical ICG points. The loop is then closed to receive any new information such as any kind of ICG subtype. At this stage, the training is completed, and the model can forecast the future inputs of the time series of the ICG subtypes.

### 4.2 Performance of the predictive model NARX

The accuracy results in Table 3 are satisfactory; the obtained RMSE values are all lower than 0.1, which is an indication of excellent machine learning performance (Raschka and Mirjalili, 2019). In this study, we notice that the minimum value of RMSE is approximately 0.003. An RMSE value of approximately 0.003 in ICG analysis is generally considered to be a highly accurate and precise result, as presented in other works in the same area studying the ICG signal that 0.003 is an indication of excellent performance (Guinot et al., 2019).

The highest RMSE in Table 4 was for subtype ABEXYOZ3; it was equal to 0.1, the threshold for excellent performance.

The distribution of the VEcv of our model showed that the maximum is 76% during the training in the open loop. The results drop down around −0 to −4% for forecasting during the closed loop; however, while the delays are removed, the VEcv of up to a maximum of 75% is observed (see Table 3).

Using the validation datasets per subtype, we notice that the minimum of VEcv was at 55%, 65% at ABEXYOZ3, and 59% and 63% at ABEXYOZ4 for datasets S and V, respectively (see Table 4). The same noticeable minimum values of Lin’s CCCs were 0.67 and 0.78 and 0.69 and 0.73 for ABEXYOZ3 and ABEXYOZ4 in datasets S and V, respectively (see Table 6).

Considering the thresholds presented in a study on 296 applications of 70 predictive models (Li, 2016; Li, 2017), the VEcv value and the performance of the models are suggested as follows:

• Very poor if VEcv ≤10%
• Poor if 10%< VEcv≤ 30%
• Average if 30%< VEcv ≤50%
• Good if 50< VEcv ≤80%
• Excellent if VEcv >80%

Thus, our predictive model falls in the good performance category with a range of 55%< VEcv ≤76%.

Moreover, comparing the obtained performance of the predictive model previously shown in Table 5 with the results reported in our previous work (Benouar et al., 2018), we notice that detection of the ICG characteristics points has been enhanced in every waveform subtype.

From Figure 14, we notice in all the ABEXYOZ complexes that with the built predictive model, it is possible to predict the ICG characteristic points in all the typical complexes at 88% for dataset S and 75% for dataset V.

The NARX predictor increases the detection of ICG points up to an effective rate of 88% for dataset S and 75% for dataset V. The effective rate is calculated after removing the number of ICG complexes with subtype ABEXYOZu, which does not contain any ICG characteristic points to detect (Benouar et al., 2018), a significant improvement compared to automatic detection. Without using NARX prediction, only the typical ABEXYOZ complexes allowed for relatively direct detection of the characteristic points; as a result, from all the ICG complexes (typical and atypical), detection was possible in only 21% of the cases for dataset S and 30% for dataset V (Benouar et al., 2018).

The results in Table 5 show the quality of forecasting the ICG characteristic points since it mainly focuses on the most variate segment of the ABEXYOZ complex where a higher number of missed points was previously noticed in the characteristic points X, Y, O, and Z. Thus, it can be observed in this study that there is a significant enhancement of detecting ICG points previously undetected.

### 4.3 Selection of X in ABEXYOZ subtypes with multiple candidates

The ABEXYOZ subtype that has more similarities with the typical ICG complex is ABEXYOZ2; however, this subtype has two X candidates (X1 or X2). Thus, this model approach enables a successful evaluation to find what is the best suitable point among X1 and X2 to be the actual X used in the calculation of LVET.

Since the model was trained with the typical ICG waveform (ABEXYOZ0), it is trained to detect the true X. In Figure 13, we can notice that the standard deviation between the target and the predicted X is less in X2 compared to X1, and it is the opposite for dataset S, as shown in Figure 13B. From the literature, X1 is considered as an X when the X2 waveform is non-pronounced. The results suggest a dependency on the recorded ICG measurements, which makes the NARX model a useful tool to evaluate the ICG subtypes and provide information to select more accurate, dynamic prediction, which leads to accurate detection of characteristic points in the different ABEXYOZ subtypes according to the datasets used.

## 5 Conclusion and future remarks

In this work, a predictive model customized specially for the different ABEXYOZ subtypes of the ICG recordings was built. The model fit data acquired with different acquisition devices. The model was created using a recurrent artificial neural network, where the typical ICG waveforms ABEXYOZ0 were used to train the model. Thus, the trained model can predict the right position of the characteristic points in the other ABEXYOZ subtypes where the position is unclear, including selection of the correct X when the ABEXYOZ subtypes present two potential Xs (X1 and X2).

Implementing NARX would enable the possibility to set up an algorithm to select the X point among candidates in each subtype, allowing for a personalized approach. This possibility will be studied further.

Knowing that without the NARX predictive model, we could detect only 21% for dataset S and 30% for dataset V, the result of applying NARX increases the detection rate of ICG points to 88% for dataset S and 75% for dataset V.

The next step is to evaluate in depth the impact of this newly increased detection of characteristic ICG points in the calculation of LVET times. For such a task, in addition to ICG recordings, thermodilution measurements will be required to allow for a proper comparative analysis.

## Data Availability

The datasets presented in this article are not publically ready available due to compliance with the data privacy preservation description included in the ethical approval. Requests to access the datasets should be directed to corresponding authors. Data sharing is possible under a collaborative framework defined by the corresponding collaboration agreement.

## Appendix A

Accuracy parameter equation.

**TABLE A1:** Accuracy parameters.

Definition	Equation
**MSE**, mean squared error: It measures the average squared difference between the predicted and actual values of the target variable. Lower values of MSE indicate better performance, with 0 being the ideal value (which indicates perfect prediction)	$MSE=1/n\;*\;\sum_{i=1}^{n}\left(yi-ŷi\right)²$ n: the number of data points, yi: the actual value of the target variable for the *i*th data point, and ŷi: the predicted value of the target variable for the *i*th data point
**RMSE**, root mean squared error: It takes the square root of the average squared difference between the predicted and actual values	RMSE = sqrt (MSE)
**MAE**, mean absolute error: It is a common metric used to evaluate the performance of a regression model. It measures the average absolute difference between the predicted and actual values of the target variable	$MAE=1/n\;*\;\sum_{i=1}^{n}\left|yi-ŷi\right|$
**VEcv**, variance error of cross-validation: It is a metric used to estimate the prediction error of a regression model using cross-validation	$VEcv=\left(1-\left(\frac{\sum_{i=1}^{n}{\left(yi-ŷi\right)}^{2}}{\sum_{i=1}^{n}{\left(yi-\bar{y}i\right)}^{2}}\right)\right)*\;100$ n is the number of observations in a validation dataset, yi is the observed value in the validation data, ŷi is the predicted value, and $\bar{y}i$ is mean of the observed values

## Appendix B

Regression plots result examples.
